# A chemical-genetic strategy reveals distinct temporal requirements for SAD-1 kinase in neuronal polarization and synapse formation

**DOI:** 10.1186/1749-8104-3-23

**Published:** 2008-09-22

**Authors:** Joanne SM Kim, Brendan N Lilley, Chao Zhang, Kevan M Shokat, Joshua R Sanes, Mei Zhen

**Affiliations:** 1 Department of Molecular Genetics, University of Toronto, Toronto, Ontario, M5S 1A8, Canada; 2 Samuel Lunenfeld Research Institute, Mount Sinai Hospital, Toronto, Ontario, M5G 1X5, Canada; 3 Department of Molecular and Cellular Biology and Center for Brain Science, Harvard University, Cambridge, MA 02138, USA; 4 Howard Hughes Medical Institute and Department of Molecular and Cellular Pharmacology, University of California, San Francisco, CA 94143, USA

## Abstract

**Background:**

Neurons assemble into a functional network through a sequence of developmental processes including neuronal polarization and synapse formation. In *Caenorhabditis elegans*, the serine/threonine SAD-1 kinase is essential for proper neuronal polarity and synaptic organization. To determine if SAD-1 activity regulates the establishment or maintenance of these neuronal structures, we examined its temporal requirements using a chemical-genetic method that allows for selective and reversible inactivation of its kinase activity *in vivo*.

**Results:**

We generated a PP1 analog-sensitive variant of SAD-1. Through temporal inhibition of SAD-1 kinase activity we show that its activity is required for the establishment of both neuronal polarity and synaptic organization. However, while SAD-1 activity is needed strictly when neurons are polarizing, the temporal requirement for SAD-1 is less stringent in synaptic organization, which can also be re-established during maintenance.

**Conclusion:**

This study reports the first temporal analysis of a neural kinase activity using the chemical-genetic system. It reveals that neuronal polarity and synaptic organization have distinct temporal requirements for SAD-1.

## Background

An emerging theme from recent studies of neural development is that many genes are employed repeatedly by different developmental processes. For example, morphogens such as WNTs are important not only for neural patterning but also for synaptogenesis [1,2]. Similarly, secreted factors such as UNC-6/netrin and semaphorins are required for both axon guidance and synapse formation [3-7], and gamma-protocadherins promote both neuronal survival and synapse formation [8,9]. These molecules may serve multiple functions through a single or multiple genetic pathways during the different developmental processes.

Distinguishing the multiple roles of a gene in neuronal development is often challenging because the developmental processes are highly interdependent: neurons that fail to polarize cannot form functional synapses, and conversely, failure in establishing synapses may lead to axon retraction and subsequent abnormalities in axon growth. Therefore, the involvement of a gene in later differentiation stages could be masked by an early-stage arrest. Addressing this issue with conventional genetic methods is not always possible. Gene knock-outs lead to an irreversible, often complete loss of gene function, and their mutant phenotypes are likely to reveal only the earliest roles during development. Temperature sensitive (*ts*) or partial loss-of-function (LOF) genetic alleles are a commonly used alternative. However, *ts *alleles are available for only a very small number of genes, and partial LOF alleles may carry sufficient residual activity to obscure the functional identification. The conditional Cre-Lox recombination-induced gene knock-out system allows temporal and tissue-specific gene inactivation; but few Cre lines permit the tight temporal control required to analyze the neuronal differentiation events that transition from one stage to the next within a narrow window of time. Therefore, a complete but reversible inactivation strategy that allows for tight temporal control and tissue specificity would be an ideal approach to addressing this issue [10].

**Figure 1:**
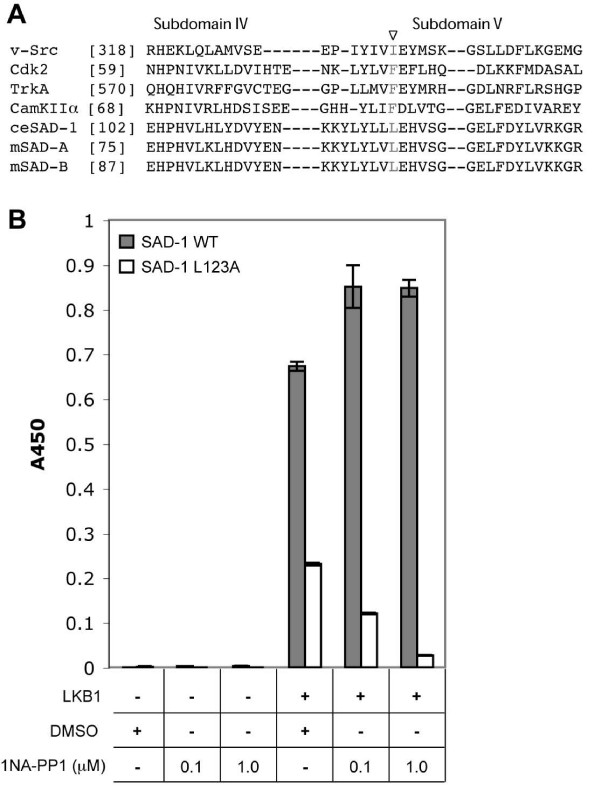
**L123A SAD-1 is functional and can be inactivated with a PP1 analog *in vitro*.** (A) Alignment of *C. elegans *SAD-1 and murine SAD kinases with other kinases successfully engineered using the chemical-genetic strategy. The gate-keeper residue is indicated in red. (B) *In vitro *kinase assay for SAD-1 activity (described in Materials and methods). In the absence of LKB1, both wild-type (WT) and analog-sensitive (*as*) versions of SAD-1 were inactive. Following treatment with LKB1, both versions became active towards tau [S262], but the kinase activity of the L123A SAD-1 was reduced 70%. Inclusion of 1NA-PP1 at the concentrations indicated caused significant inhibition of SAD-1*as *but not of wild-type SAD-1. ***p < 0.001, paired *t*-test, between DMSO- and 1NA-PP1-treated L123A SAD-1 samples. The y-axis indicates signal from ELISA assay for phospho-tau [S262]. Error bars indicate the standard error of the mean.

A chemical-genetic method that combines the specificity of genetics with the reversibility and temporal control of pharmacology has been developed for kinases. Initially identified as an inhibitor for the Src kinase [11], the adenine analog PP1 was modified to selectively bind genetically sensitized kinases [12]. In this method, the hydrophobic gate-keeper residue in the ATP-binding pocket of a kinase of interest is mutated to glycine or alanine (Figure 1A), creating extra space in the ATP-binding pocket. Exposing this mutant kinase to membrane-permeable, non-hydrolyzable PP1 analogs containing a bulky moiety (for example, 1NA-PP1) allows for selective inactivation of the kinase of interest [13-19].

In this study, we adopted this chemical-genetic approach to study the temporal requirements for a serine/threonine kinase, SAD-1 (Synapses of amphids defective-1). Initially identified in *Caenorhabditis elegans*, SAD-1 is required for proper neuronal polarity and synaptic organization [20,21]. In *sad-1 *LOF mutants, synaptic vesicles and other presynaptic proteins are trafficked to both axons and dendrites, and vesicle clusters are abnormally diffuse at synapses. Its mammalian orthologs, SAD-A and SAD-B, also play multiple roles in neural development. Mouse SAD-A and SAD-B (also known as BRSK2 and BRSK1, respectively) function redundantly to regulate neuronal polarity *in vivo *[22,23], and SAD-B regulates the release of synaptic neurotransmitters in cultured rat neurons [24].

The mechanism of SAD-1-mediated regulation of neuronal polarity and synaptic organization is not well-understood. Our recent study showed that SAD-1 physically interacts with a scaffolding protein, NAB-1 (Neurabin-1), and that this interaction is essential for regulating neuronal polarity but not synaptic organization [21]. These data suggest that SAD-1 regulates neuronal polarity through pathways that are distinct from those for synaptic organization. Whether SAD-1 is required for the establishment or maintenance of the two neuronal structures is unknown. To dissect the temporal requirements for SAD-1 in neuronal polarity and synaptic organization, precise temporal inactivation of SAD-1 activity *in vivo *is needed. The kinase activity of SAD proteins is essential for their *in vivo *functions [20,23], making SAD-1 an ideal candidate for a chemical-genetic system that allows for inducible and reversible inactivation of kinase activity.

The chemical-genetic strategy has been used in yeast, *Arabidopsis*, *Drosophila*, mammalian cell lines, and mice [13-19]. Here we report the first successful application of this method in *C. elegans*. Using this system, we discovered that the kinase activity of SAD-1 is required at developmental stages that coincide with the establishment of neuronal polarity and synaptic organization. However, while inactivating SAD-1 when neurons are establishing polarity and synapses led to irreversible defects in neuronal polarity, defects in synaptic organization were reversible and corrected by SAD-1 activity during maintenance. Therefore, these differentiation events have distinct temporal requirements for SAD-1 activity.

## Results

### L123A SAD-1 is functional and can be inactivated by a PP1 analog *in vitro*

To generate a SAD-1 variant that is sensitive to PP1 analogs, its gate-keeper residue, leucine 123, was mutated to an alanine (L123A; Figure 1A). The kinase activity of L123A SAD-1 was compared to that of the wild-type enzyme in an *in vitro *kinase assay using tau as a substrate [23] (Figure 1B). Upon activation by the LKB1 complex [25], both wild-type and L123A mutant enzymes were able to phosphorylate tau at S262. L123A SAD-1 was 30% as active as the wild-type enzyme even at the highest ATP concentrations tested (Figure 1B). An alteration in activity of this magnitude is not uncommon for kinases with mutated gate-keeper residues, and in most cases the decrease in activity does not affect the ability of the kinase to properly function *in vivo *[13].

We next tested the sensitivity of wild-type and L123A SAD-1 to a PP1 analog, 1NA-PP1. The wild-type enzyme was unaffected by 1NA-PP1, but L123A SAD-1 was inhibited in a dose-dependent manner (Figure 1B). In summary, L123A SAD-1 retains kinase activity and is selectively sensitive to a PP1 analog.

### L123A SAD-1 is functional *in vivo*

To determine if L123A SAD-1 can substitute for wild-type SAD-1 *in vivo*, we asked whether it can rescue neuronal polarity and synaptic organization phenotypes of *sad-1 *null mutants. The L123A mutation was introduced to a *sad-1 *construct that rescues defects in both neuronal phenotypes of *sad-1 *mutants [20,21]. This construct, *P*_*sad-1*_-*sad-1*(*L123A*), was introduced into *sad-1 *(*ky289*) null mutants along with *juIs1*, a fluorescent marker of synaptic vesicles in GABAergic neurons (*P*_*unc-25*_-*snb-1*::*gfp*) [26]. The *juIs1 *marker has been shown to be a reliable reporter for neuronal polarity [21] and synaptic organization [20,21].

**Figure 2:**
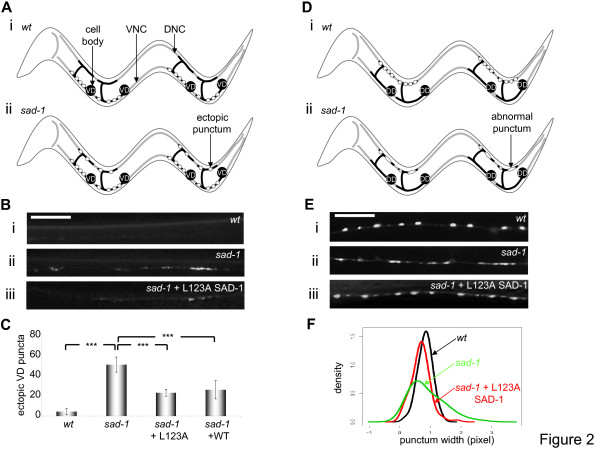
**L123A SAD-1 is functional *in vivo*. (A, B) Polarity phenotype.** Axons of VD neurons innervate ventral body wall muscles, forming presynaptic terminals exclusively on the ventral side (Ai). In wild-type (*wt*) animals, only few, if any, ectopic *juIs1 *puncta are observed on the dorsal side (Bi). In *sad-1 *LOF mutants, many ectopic *juIs1 *puncta are found (Aii, Bii). Expressing L123A SAD-1 partially rescued this phenotype, and fewer ectopic puncta were observed (Biii). DNC, dorsal nerve cord. VNC, ventral nerve cord. (C) Quantification of polarity phenotypes. Polarity defects were quantified by counting the number of ectopic *juIs1 *puncta per animal. Expressing L123A SAD-1 in *sad-1 *mutants reduced their polarity defect more than 50%, similar to expressing wild-type SAD-1. ***p < 0.001, Wilcoxon rank-sum test. Error bars indicate standard deviations. (D, E) Synaptic organization phenotype. In wild-type animals, *juIs1 *puncta in DD neurons appear round and discrete along the dorsal nerve cord (Di, Ei). In *sad-1 *mutants, diffuse or smaller puncta are observed (Dii, Eii). Expression of L123A SAD-1 rescued this phenotype and restored the *juIs1 *morphology (Eiii). (F) Quantification of synaptic organization phenotypes. Kernel density estimates of the punctum widths show a much wider distribution of punctum widths (that is, reduced kurtosis) in *sad-1 *mutants compared to wild-type animals. L123A SAD-1 rescued this *sad-1 *defect. Scale bar, 5 μm.

We first examined if L123A SAD-1 rescued the neuronal polarity defect in *sad-1 *mutants. In adult animals, the two classes of *C. elegans *GABAergic motoneurons, DDs and VDs, innervate dorsal and ventral body wall muscles, respectively. We observed vesicle trafficking in VD neurons by selectively eliminating the *juIs1 *expression in DD neurons (see Materials and methods). The polarity of VD neurons is reflected by the selective accumulation of the presynaptic *juIs1 *vesicle clusters, or puncta, in axonal processes along the ventral nerve cord and their exclusion from dendrites along the dorsal nerve cord in wild-type animals as previously described [21,27] (Figure 2Ai). In *sad-1 *mutants, on the other hand, vesicles are present in both ventral axons and dorsal dendrites (Figure 2Aii). This polarity defect was quantified by counting the number of ectopic, dorsal *juIs1 *puncta (Figure 2B, C). As previously reported [21], a 10-fold increase in ectopic *juIs1 *puncta was observed in *sad-1 *mutants relative to wild-type animals (*p *= 3.2 × 10^-6^). In the L123A SAD-1-expressing *sad-1 *mutants, the number of ectopic *juIs1 *puncta was decreased more than 50% (*p *= 3.2 × 10^-6 ^compared to *sad-1 *mutants; Figure 2B, C). This partial rescue may reflect the reduced kinase activity of L123A SAD-1 compared to the wild-type enzyme (Figure 1B). It is noteworthy, however, that wild-type SAD-1 also only partially rescued polarity defects in *sad-1 *null mutants (*p *= 3.2 × 10^-5 ^compared to *sad-1 *mutants; Figure 2C) [21], suggesting that the rescue of the *sad-1 *polarity phenotype is sensitive to the protein level typically over-expressed by transgenes.

We also asked whether L123A SAD-1 could rescue the synaptic organization defect in *sad-1 *mutants. In wild-type animals, *juIs1 *puncta in DD axons were round and discrete along the dorsal nerve cord, displaying the characteristic 'beads-on-a-string' morphology (Figure 2Di, Ei). In *sad-1 *mutants, *juIs1 *puncta appeared diffuse or smaller (Figure 2Dii, Eii). This defect in the *juIs1 *morphology correlates with a more diffuse distribution of synaptic vesicles in *sad-1 *mutants as shown by electron microscopy [20]. For a quantitative analysis of the *juIs1 *morphology, the width of each punctum was measured, and the population distribution of punctum widths was examined. As shown in Figure 2F, *sad-1 *mutants displayed a broader distribution of punctum widths, or 'lower kurtosis' of the distribution curve, than wild-type animals, consistent with the appearance of both diffuse and smaller puncta. In the L123A SAD-1-expressing *sad-1 *mutants, *juIs1 *puncta appeared more round and discrete (Figure 2E, F). Thus, L123A SAD-1 rescues synaptic organization as well as polarity defects in *sad-1 *mutants.

### L123A SAD-1 can be selectively inactivated by 1NA-PP1 *in vivo*

**Figure 3:**
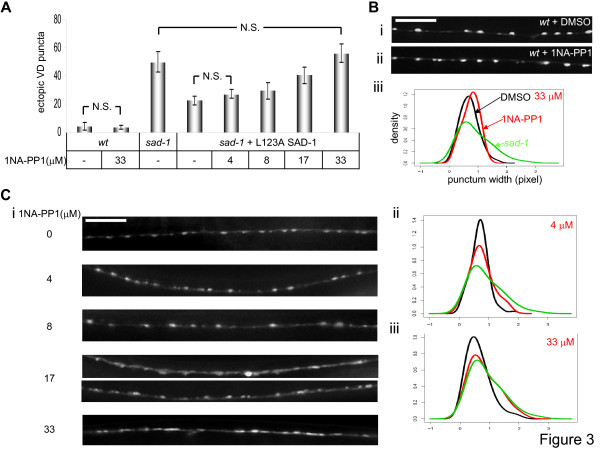
**1NA-PP1-induced SAD-1*as *inactivation is dose-dependent.** (A) Dose-response in polarity phenotype. L123A SAD-1-expressing *sad-1 *animals were exposed to various concentrations of 1NA-PP1, and the number of ectopic *juIs1 *puncta on the dorsal side of each animal was counted. At 4 μM, the level of polarity defect was similar to the animals exposed to DMSO. At 8 μM and 17 μM, the inhibiting effects were stronger but incomplete. At 33 μM, complete inhibition of SAD-1*as *was observed. 1NA-PP1 had no effect on the polarity phenotype of wild-type (*wt*) animals. N.S., not significant, Wilcoxon rank-sum test. Error bars indicate standard deviations. (B, C) Dose-response in synaptic organization phenotype. At 33 μM, 1NA-PP1 had no effect on the *juIs1 *morphology of wild-type animals (compare Bi and ii; quantified in Biii). L123A SAD-1-expressing *sad-1 *animals were exposed to various concentrations of 1NA-PP1, and the *juIs1 *morphology on the dorsal side of the animals was observed (Ci). At 4 μM and 8 μM, most animals appeared normal. At 17 μM, while some *juIs1 *puncta appeared defective (upper panel), others were normal (lower panel). At 33 μM, the rescuing effects of SAD-1*as *were inhibited in all animals. Quantification and density estimates of the punctum widths show that while 4 μM 1NA-PP1 had little effect (Cii), 33 μM 1NA-PP1 completely inhibited L123A SAD-1 (Ciii). All density graphs show punctum widths on the x-axis and density on the y-axis. Scale bar, 5 μm.

Next we asked whether L123A SAD-1 could be selectively inactivated by 1NA-PP1 *in vivo*. We devised a liquid culture method using 96-well tissue culture dishes (Additional file 1), which allows for effective entry of 1NA-PP1 into animals through ingestion and also reduces the quantity of analog required. A dose-response curve was generated by exposing animals to different concentrations of 1NA-PP1 throughout their life and then assaying for polarity and synaptic organization phenotypes in adults (Figure 3). Whereas only partial inhibition was observed at lower concentrations, complete inhibition was observed at 33 μM 1NA-PP1 (*p *= 0.072 compared to *sad-1 *mutants; Figure 3A, C), and this concentration was used subsequently. This is comparable to, albeit slightly higher than, concentrations used for *in vivo *inactivation of PP1 analog-sensitive mammalian kinases [14,16]. Wild-type and L123A SAD-1-expressing *sad-1 *animals exposed to 33 μM 1NA-PP1 displayed slightly slower, but otherwise normal, growth. Exposing L123A SAD-1-expressing *sad-1 *animals to vehicle DMSO (Figure 3A, Ci) or treating wild-type animals with 1NA-PP1 (Figure 3B) had no effect on neuronal polarity or synaptic organization.

These data show that L123A SAD-1 is both functional and selectively inactivated by 1NA-PP1 *in vivo*. We therefore hereafter refer to L123A SAD-1 as an analog-sensitive version of SAD-1 kinase (SAD-1*as*) and L123A SAD-1-expressing *sad-1 *mutants as SAD-1*as *animals.

### SAD-1 kinase activity is required for the establishment of neuronal polarity and synaptic organization

**Figure 4:**
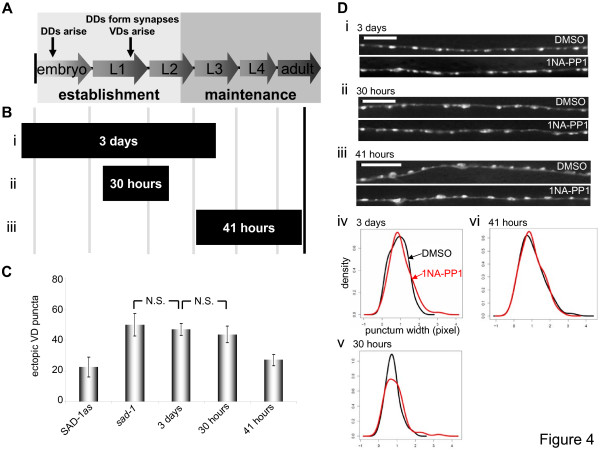
**SAD-1 kinase is required during the establishment of neuronal polarity but largely dispensable for the maintenance of neuronal polarity and synaptic organization.** (A) Development of the GABAergic motoneurons. DD neurons arise embryonically and form synapses in late L1. VD neurons arise concomitantly. Whereas the establishment of DD and VD neurons is mostly complete by the end of L2, they are maintained for the *C. elegans *lifetime. (B) Two early larval and one late exposure. SAD-1*as *animals were exposed to 1NA-PP1 throughout establishment (Bi), partially during establishment (Bii), or during maintenance (Biii) (filled bars) and observed at an adult stage (solid line). (C) Polarity phenotypes following the exposures. The three-day establishment exposure abolished the rescuing effects of SAD-1*as*, and the 30-hour duration was sufficient to replicate this full establishment exposure. The 41-hour exposure had no effect. N.S., not significant, Wilcoxon rank-sum test. Error bars indicate standard deviations. (D) Synaptic organization phenotypes following the exposures. Animals treated with 1NA-PP1 throughout establishment for three days occasionally displayed diffuse *juIs1 *puncta (bottom panel) compared to DMSO-treated animals (upper panel) (Di). The 30-hour and 41-hour durations had no effect on the SAD-1*as*-induced rescue (Dii-iii). Quantification and density estimates of the punctum widths show that there is no obvious difference between DMSO (black) and 1NA-PP1 (red) treatments for any of the three exposures (Div-vi). All density graphs show punctum widths on the x-axis and density on the y-axis. Scale bar, 5 μm.

Embryonically born DD neurons establish synapses with dorsal body wall muscles at the end of the first larval (L1) stage. VD neurons arise shortly thereafter and establish synapses with ventral body wall muscles [28] (Figure 4A). By the end of the second larval (L2) stage, polarization and synaptogenesis in these motoneurons are nearly complete. New synapses are added after the L2 stage, but they constitute less than 20% of the total number of synapses in adult animals [29]. We therefore defined the transition from the L2 stage to the L3 stage as the end of establishment and the beginning of maintenance for both polarity and synaptic organization in VD and DD neurons, respectively.

As noted above, life-long exposure of SAD-1*as *animals to 1NA-PP1 led to defects in neuronal polarity and synaptic organization (Figure 3). To ask whether the kinase activity of SAD-1 is required for the establishment or maintenance of neuronal polarity, we exposed SAD-1*as *animals to 1NA-PP1 during early larval stages or later (Figure 4B) and counted ectopic *juIs1 *puncta in adults (Figure 4C). Inhibiting SAD-1*as *throughout establishment and not maintenance was sufficient to abolish its rescuing effects on the polarity defect (Figure 4Bi, C). Moreover, inhibiting SAD-1*as *for as few as 30 hours during L1 and L2 was sufficient to elicit the same level of polarity defect (Figure 4Bii, C). Conversely, an exposure to 1NA-PP1 throughout maintenance had little effect on the polarity phenotype of SAD-1*as *animals (Figure 4Biii, C). These results suggest that SAD-1 activity is required for the establishment, but not the maintenance, of neuronal polarity.

In contrast to the dramatic effects of early larval inhibition on adult neuronal polarity, inactivating SAD-1*as *for either three days or 30 hours in early larvae had little effect on the *juIs1 *morphology in adults (Figure 4B, D). Diffuse puncta were observed occasionally after three days of inactivation (Figure 4Di), but this subtle abnormality was not quantitatively significant (Figure 4Div).

**Figure 5:**
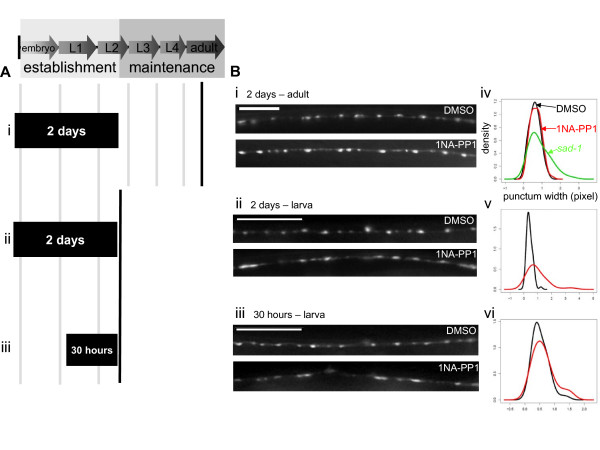
**SAD-1 kinase establishes synaptic organization.** (A) Developmental exposures. SAD-1*as *animals were exposed to 1NA-PP1 throughout establishment (Ai-ii) or partially during establishment (Aiii) (filled bars). Observations were made at an adult stage (Ai) or immediately following the exposures (Aii-iii) (solid lines). (B) Synaptic organization phenotypes following the exposures. The two-day establishment exposure to 1NA-PP1 had no effect on the SAD-1*as*-induced rescue observed at the adult stage (Bi). However, when SAD-1*as *animals were observed immediately after treatment, the rescuing effects of SAD-1*as *were abolished (Bii-iii). Quantification and density estimates of the punctum widths show that in the adult stage, DMSO (black) and 1NA-PP1 (red) treatments appeared identical and rescued compared to *sad-1 *mutants (Biv). In contrast, immediately after 1NA-PP1 treatment, obvious changes in the distribution of the punctum widths were observed (Bv-vi). All density graphs show widths on the x-axis and density on the y-axis. Scale bar, 5 μm.

These results suggest at least two possibilities. SAD-1 may be required solely for the maintenance of already-established synaptic organization and not for synapse formation. However, this is unlikely as exposing animals selectively during maintenance did not affect the *juIs1 *morphology (Figure 4Biii, Diii, vi). Alternatively, SAD-1 kinase activity during the maintenance stage may be sufficient to re-establish synaptic organization. To test this possibility, we treated SAD-1*as *animals with 1NA-PP1 either throughout establishment until the end of the L2 stage or during the short 30-hour duration of DD synapse formation and observed their *juIs1 *morphology immediately after treatment (Figure 5Aii, iii). Indeed, *juIs1 *puncta appeared diffuse immediately after treatment, suggesting that inhibiting SAD-1*as *during synaptogenesis abolished its rescuing effects on vesicle clustering (Figure 5B). Therefore, the re-activation of SAD-1*as *during maintenance following the early exposures to 1NA-PP1 led to restoration of the *juIs1 *morphology in adults (Figures 4Dii–iii, 5Bi). This phenomenon was specific to synaptic organization and not neuronal polarity, as early larval exposures to 1NA-PP1 followed by reactivation of SAD-1*as *did not correct the vesicle trafficking defect (Figure 4C). Thus, while SAD-1 activity is required strictly when neurons are polarizing, it can promote synaptic organization during both establishment and maintenance.

### Distinct temporal requirements exist for SAD-1 kinase activity during neuronal development

Our study demonstrates the first application of the chemical-genetic system in *C. elegans*. To further test our findings from using this new methodology, we employed an independent method. Instead of inactivating SAD-1, we utilized a heat-shock (HS) inducible system to express wild-type SAD-1 in *sad-1 *null mutants. If SAD-1 determines neuronal polarity solely in developing neurons, expression of SAD-1 after the L2 stage would not rescue the neuronal polarity defect in adults. In contrast, if SAD-1 could re-establish synaptic organization in the maintenance stage, expression of SAD-1 after the L2 stage should rescue the synaptic morphology defect in adults.

**Figure 6:**
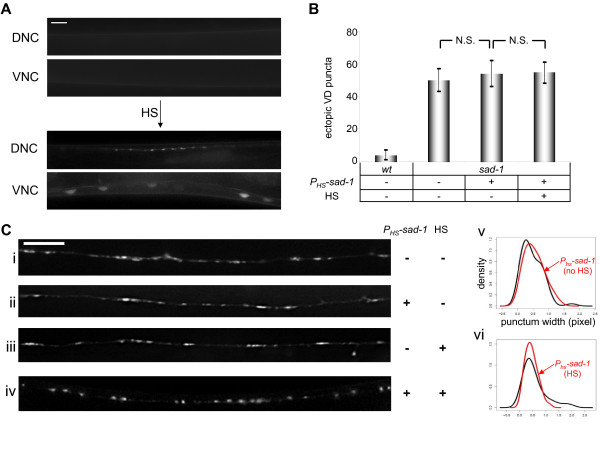
**Expression of SAD-1 in *sad-1 *mutants during maintenance rescues defects in synaptic organization but not in neuronal polarity.** (A) GFP::SAD-1 expression in *C. elegans *nervous system following heat shock (HS). No GFP was detected in animals reared at 22°C (top panels). However, upon HS, strong GFP fluorescence was detected in various neurons, including those along the body (bottom panels). GFP fluorescence was seen along processes on the dorsal nerve cord (DNC) as well as in cell bodies on the ventral nerve cord (VNC). (B) Polarity phenotype following post-L2 expression of SAD-1. Expression of SAD-1 during maintenance had no rescuing effects on the VD polarity phenotype. N.S., not significant, Wilcoxon rank-sum test. Error bars indicate standard deviations. (C) Synaptic organization phenotype following post-L2 expression of SAD-1. Diffuse *juIs *puncta were observed in *sad-1 *mutants before (Ci) and after (Ciii) HS. Expression of SAD-1 during maintenance rescued the *juIs1 *morphology phenotype (compare Cii and Civ). Quantification and density estimates of the punctum widths show no differences between *sad-1 *mutants (black) and HS construct-containing mutants (red) (Cv). However, upon HS, changes in the distribution of punctum widths can be observed (Cvi). Both density graphs show widths on the x-axis and density on the y-axis. Scale bar, 5 μm.

We used a promoter that drives ubiquitous expression in *C. elegans *upon HS (pPD118.26) [30]. To confirm its expression in the nervous system and examine the stability of SAD-1, an amino-terminal green fluorescent protein (GFP) fusion of SAD-1 under the HS protein promoter (*P*_*HS*_*-gfp*::*sad-1*) was generated. Only after HS was strong GFP expression observed, confirming that GFP::SAD-1 expression was tightly regulated by temperature in our experimental system (described in Materials and methods). In the nervous system, GFP::SAD-1 expression was observed along the processes and in the cell bodies of body neurons (Figure 6A).

**Figure 7:**
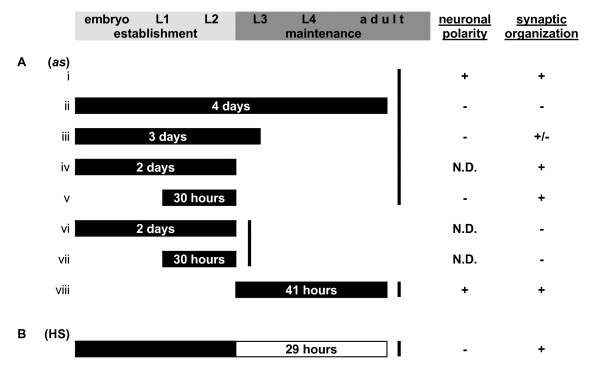
**Summary of neuronal phenotypes following different temporal exposures to 1NA-PP1 or heat shock**. (A) 1NA-PP1 exposures of SAD-1*as *animals (italics). (B) Heat shock expression of wild-type SAD-1 in *sad-1 *mutants (bold). Filled bars indicate no SAD-1. Solid lines represent points of observation. +, normal; -, defective; N.D., no data. Slower growth was observed in liquid culture than on agar (compare Aviii and B), likely caused by lower levels of oxygen in liquid.

We generated a HS-inducible SAD-1 construct (*P*_*HS*_*-sad-1*) and transformed it into *sad-1 *(*ky289*) null mutants. SAD-1 was expressed by HS at the end of the L2 stage, and the polarity and synaptic organization phenotypes were observed in adults (Figure 6B, C). No rescue in the polarity defect was observed (*p *= 0.51 between with and without HS; Figure 6B). In contrast, following HS, the *juIs1 *morphology was rescued, appearing more round and discrete (Figure 6Cii, 6iv). This was not a result of HS treatment itself as the *juIs1 *morphology in *sad-1 *mutants was unaltered by HS (Figure 6Ci, iii). Therefore, consistent with our findings from the chemical-genetic system, the expression of SAD-1 in the maintenance stage rescued synaptic organization but not neuronal polarity defects (Figure 7).

## Discussion

We report here the first successful application of the chemical-genetic system to analyzing the temporal requirements of a kinase in *C. elegans*. A PP1 analog-sensitive version of SAD-1 kinase, SAD-1*as*, was functional and sensitive to 1NA-PP1-mediated inhibition, both *in vitro *and *in vivo*. Using this system, we temporally inactivated SAD-1 kinase during different stages of the *C. elegans *life-cycle and found that SAD-1 activity is necessary in establishing both neuronal polarity and synaptic organization but dispensable for their maintenance (Figure 7). However, while SAD-1 activity is required strictly during a narrow window of time to establish neuronal polarity, its activity is not temporally restricted to the establishment stage and sufficient to re-establish synaptic organization in later stages. Our study thus reveals temporally distinct requirements for SAD-1 activity during neuronal differentiation.

### A chemical-genetic approach in analyzing kinase functions in *C. elegans*

We showed that SAD-1*as *can be generated by mutating the gate-keeper residue, allowing inducible, fast, and reversible inhibition of its kinase activity with a specific small-molecule inhibitor. In this study, a small-volume liquid culture system was devised that offered several advantages over conventional plate cultures (Additional files 1 and 2; described in Materials and methods). The small volumes of the liquid culture required only microgram quantities of 1NA-PP1, reducing the quantity of inhibitor used 30-fold. The liquid culture also facilitated efficient administration of the membrane-permeable inhibitor through feeding.

PP1 analogs effectively blocked the activity of *as*-kinases at nanomolar concentrations in yeast and mammalian cell cultures [13,16]. In our study, micromolar-range 1NA-PP1 was required to achieve complete inhibition of SAD-1*as *(Figure 3). This is likely caused by the poor permeability of *C. elegans *cuticles, only allowing entry through feeding. Micromolar concentrations of PP1 analogs were also required to inhibit mammalian *as *kinase activities in intact animals through oral administration [14,16]. Therefore, the efficacy of 1NA-PP1 in *C. elegans *is comparable to that in mice *in vivo*.

The kinetics of 1NA-PP1 entry in *C. elegans *is unknown. However, we do not think slow kinetics of entry was a confounding factor in our analyses because the shortest exposure time of 30 hours was sufficient for 1NA-PP1 entry and SAD-1*as *inactivation (Figure 7Av, vii). Indeed, a similar PP1 analog, 1NM-PP1, has been shown to cross the murine blood-brain barrier within minutes [14]. We also do not believe differential susceptibilities to 1NA-PP1 entry existed during different larval stages of the *C. elegans *life-cycle because the phenotypic difference in synaptic organization between the lifetime exposure (Figure 7Aii) and early larval exposures (Figure 7Avi, vii) suggests that there was sufficient 1NA-PP1 entry and inhibition of SAD-1*as *across the larval stages.

Although the chemical-genetic system has proven to be quite a versatile and effective tool for regulating the activity of different kinases in yeast and cell culture studies, its application to intact animal models has been limited. Many kinase families display strong functional conservation across different animal species. The ease of genetic manipulation, simple physiology, and short developmental cycle make *C. elegans *an attractive target for using chemical genetics to temporally dissect the functional requirements of kinases. Our protocol may be applicable to other kinases or even other proteins containing ATP-binding domains [31] in *C. elegans*.

### Distinct temporal requirements for SAD-1 activity in neuronal polarization and synapse formation

We previously demonstrated that SAD-1 regulates neuronal polarity and synaptic organization through different genetic pathways [21]. Using inducible modulation of protein activity (*as*) and of mRNA expression (HS), we have now demonstrated that the neuronal structures also display different temporal requirements for SAD-1 activity.

The molecular basis for the temporal distinction between neuronal polarity and synaptic organization is unknown. The simplest explanation is that SAD-1 targets different substrates during polarization and synapse formation. While the substrates involved in neuronal polarization may only be present or active during the establishment stage, those involved in synapse formation may be present throughout establishment and maintenance. The mammalian orthologs of SAD-1, SAD-A and SAD-B, have been shown to regulate neuronal polarity through phosphorylation of tau, a microtubule associated protein [22,23], and subsequent promotion of microtubule dynamics during axon extension [32]. As an activator of microtubule dynamics, SAD-1 would define neuronal polarity only during the establishment stage. At synapses, the diffuse vesicle clustering in *sad-1 *mutants may reflect a failure in regulating vesicle release at the active zone or vesicle retrieval at the periactive zone. SAD-1 might phosphorylate distinct substrates that are required for organizing the endo- and exo-cytosis apparatus or for facilitating the interactions between vesicles and active zone/periactive zone regions.

To further our understanding of SAD-1, it is critical to identify all of its physiological substrates. The chemical-genetic system has been used to identify substrates of kinases in *in vitro *assays [33,34]. Exploiting our SAD-1*as *system with substrate-labelling analogs may facilitate the identification of substrates and provide insights to the mechanism of SAD-1 in the future.

## Materials and methods

### Strains

All *C. elegans *strains were cultured at 22°C using standard procedures on Nematode Growth Medium [35] with OP50 *Escherichia coli *as the food source.

*juIs1 *(*P*_*unc*-25_-*snb-1*::*gfp*) has been described previously [26]. pJH685 (*P*_*sad*-1_-*sad-1*(*L123A*)) was co-injected with a *P*_*odr*-1_-*gfp *marker into *sad-1 *(*ky289*); *juIs1 *animals and integrated into the *C. elegans *genome by UV irradiation. The integrants were out-crossed four times against wild-type N2 animals to generate *hpIs89*. From this, *hpIs89*; *sad-1 *(*ky289*); *juIs1 *was generated and termed 'SAD-1*as *animals'. pJH73 (*P*_*unc*-115_-*sad-1*) was co-injected with a *P*_*odr*-1_-*gfp *marker into *sad-1 *(*ky289*); *juIs1 *animals to generate *sad-1 *(*ky289*); *juIs1*; *hpEx1043 *animals. pJH1360 (*P*_*HS*_-*sad-1*) was co-injected with a *P*_*odr*-1_-*gfp *marker into *sad-1 *(*ky289*); *juIs1 *animals to generate *sad-1 *(*ky289*); *juIs1*; *hpEx1431 *animals. pJH1368 (*P*_*HS*_-*gfp*::*sad-1*) was co-injected with a *P*_*lin*-15_-*lin-15 *into *lin-15 *animals to generate *lin-15*; *hpEx1421 *animals.

### Plasmids

The L123A mutation was engineered by oligonucleotide site-directed mutagenesis in a *sad-1 *genomic DNA fragment to generate plasmid pBNL32. pJH685 (*P*_*sad*-1_-*sad-1*(*L123A*)) was generated by sub-cloning the *Nhe*I/*Xma*I fragment of pBNL32 into pJH630, which contains the minimal rescuing region (*Sac*II/*Avr*II fragment) from the cosmid F15A2. pJH73 was generated by inserting a *sad-1 *mini-gene fragment from pJH56 under the *unc-115 *promoter into the pBSK vector using the *Not*I and *Bam*HI sites. pJH1360 was generated by inserting a *sad-1 *mini-gene fragment from pJH56 into the HS pPD118.26 vector using the *Bam*HI and *Apa*I sites. pJH1368 was generated by cloning *gfp *from pJH21 into the 5' end of *sad-1 *in pJH1360 using the *Bam*HI site.

### Protein purification and kinase assays

Wild-type and L123A versions of SAD-1 cDNA were sub-cloned into pGEX6p-1. Proteins were expressed in *E. coli *BL21 by inducing with 0.1 mM isopropyl beta-D-thiogalactoside (IPTG) for 24 hours at room temperature with aeration. Purification was performed as described in [25] except that elution was performed using PreScission protease (GE Healthcare, Buckinghamshire, England) according to the manufacturer's specifications. Eluted protein was snap-frozen at -80°C until use. Due to low yields of protein, quantification and normalization of the wild-type and L123A proteins was performed by western blot using an anti-SAD-1 antibody previously described [21].

For kinase assays, an activation reaction was first performed to phosphorylate SAD-1 at the activation loop threonine, a modification required for full activity of AMP-activated protein kinase (AMPK) family members [25]. In this reaction, approximately 5 μg of wild-type or L123A SAD-1 was incubated with 100 ng of LKB1 complex (Upstate, Billerica, MA, USA) at room temperature for 30 minutes in a kinase buffer (KB) containing 25 mM Tris-HCl, 10 mM MgOAc, 1 mM dithiothreitol, 0.1% (v/v) Triton X-100, and 0.1 mg/ml bovine serum albumin, pH 7.4 plus 100 μM ATP in a volume of 50 μL. SAD-1 kinase assays were performed using purified tau as a substrate: 400 ng of purified 4R tau (a kind gift of MA Glicksman, Brigham & Women's Hospital, Cambridge, MA, USA) diluted in TBS was added to each well of a 96-well tissue culture dish (standard tissue culture treated polystyrene; Corning, Lowell, MA, USA) followed by overnight incubation at 4°C. The wells were washed with four changes of TBS with 0.1% Tween-20 (TBS-T) and were then equilibrated with KB prior to the start of the assay. Immediately prior to the start of the assay, the KB was aspirated and 10 μL of KB containing the desired concentrations of DMSO/1NA-PP1 (final DMSO concentration was 3.3%) was added. The activation reaction was diluted in the same buffer, but the ATP concentration was adjusted to 500 μM, and 20 μL aliquots were added to the wells (30 μL final reaction volume). Reactions, performed in triplicate, were incubated at room temperature and were stopped after 30 minutes by aspirating the liquid and adding 50 μL of 10 mM EDTA. To measure phosphorylation of immobilized tau at Ser262, an ELISA was performed using an anti-phosphoTau [S262] antibody (1:1000; Stressgen, Ann Arbor, MI, USA) and a horseradish peroxidase-conjugated goat anti-rabbit antibody (1:25,000; Jackson Immunoresearch, West Grove, PA, USA). Antibodies were diluted in Superblock TBS blocking solution (Pierce, Rockford, IL, USA), and bound antibody was visualized using TMB liquid chromogenic substrate (Sigma-Aldrich, St Louis, MO, USA). After sufficient signal developed, the reactions were stopped by addition of an equal volume of 1 N HCl, and the plates were read in a Spectramax 384 plate reader. Background signal was determined by measurements from multiple wells that did not contain kinase reactions.

### Observation of *juIs1 *puncta in VD neurons

As there is no VD-specific promoter, selective visualization of *juIs1 *puncta in VD neurons was achieved by inhibiting expression of the *juIs1 *marker in DD neurons as previously described [21,27]. Briefly, double-stranded RNA against the *unc-30 *transcription factor – which activates the *unc-25 *promoter used to express the *juIs1 *marker – was synthesized from pJH573 as previously described [36] and injected at 100 ng/μL into adult animals carrying the *juIs1 *marker [27]. Progenies of the injected animals that retained the GFP signal in all 13 of the VD neuron cell bodies but in none of the six DD neuron cell bodies were scored.

### DMSO and 1NA-PP1 treatments in *C. elegans*

Animals were exposed to DMSO (Sigma-Aldrich, Oakville, Ontario, Canada) or 1NA-PP1 in liquid culture. Overnight LB cultures of OP50 *E. coli *were divided into 500 μL aliquots, and pellets were collected and stored at -20°C until use. Each pellet was resuspended in 650 μL of S-medium. Animals were incubated in 150 μL of the OP50 *E. coli *resuspension containing 0.33% DMSO or 33 μM 1NA-PP1/0.33% DMSO added to the wells of a 96-well tissue culture dish (Falcon, St Louis, MO, USA). Exposures throughout the *C. elegans *life-cycle for validation studies were achieved by treating fourth larval (L4) stage animals and observing their offspring in an adult stage. The solutions were renewed every three days. Exposures until earlier than the adult stage were performed by treating L4 animals and transferring their offspring onto OP50 *E. coli*-seeded agar plates at desired stages, assessed by the stage of gonad development. Early first larval (L1) stage-arrested animals were obtained by lysing gravid adult animals in hypochlorite on unseeded agar plates and allowing them to develop at 22°C for 16–18 hours. Late second larval stage (L2) animals were recovered by transferring and keeping early L1-arrested animals on OP50 *E. coli*-seeded agar plates for 30 hours at 22°C. Detailed procedures on the incubations are described in Additional files 1 and 2.

### Heat-shock treatments

All HS treatments were performed by incubating animals at 30°C for 7 hours. A gradual decrease in the GFP::SAD-1 protein expression level, reflected by the intensity of GFP fluorescence, was observed following HS over the course of a day. Therefore, for post-L2 HS treatments, late L2 animals obtained as described above were heat-shocked twice, 15 hours apart.

### Puncta quantification and statistical analyses

Significance-testing employed paired *t*-test for the *in vitro *kinase assay and the Wilcoxon rank-sum test for the VD neuron analyses as implemented in the R statistical environment (v2.6.2) [37]. *juIs1 *puncta were quantified by an in-house developed program called 'punctaanalyser' using the MatLab software (v6.5; Mathworks, Inc., Natick, MA, USA) and analyzed using kernel density estimation implemented in R (v2.6.2).

## Abbreviations

*C. elegans*: *Caenorhabditis elegans*; GFP: green fluorescent protein; HS: heat shock; LOF: loss-of-function; SAD: Synapses of amphids defective; ts: temperature sensitive; SAD-1*as*: analog-sensitive version of SAD-1 kinase.

## Competing interests

The authors declare that they have no competing interests.

## Authors' contributions

JSMK designed and conducted *C. elegans *experiments, analyzed data, prepared figures, and wrote the manuscript. BNL conceived the study, designed and performed *in vitro *experiments, analyzed data, and prepared figures. CZ and KMS supplied 1NA-PP1. BNL, JRS, and MZ edited the manuscript. JRS and MZ supervised the work. All authors read and approved the manuscript.

## Supplementary Material

Additional file 1 Experimental system and different exposures (1). Exposures to DMSO and/or 1NA-PP1. Each flow corresponds to a time-course as indicated in square boxes. L4 animals were exposed to DMSO/1NA-PP1, and their progenies were transferred to agar plates at different stages to be observed immediately or at an adult stage. Full lifetime exposures required four days of incubation; solutions were renewed after three days. Click here for file

Additional file 2 Experimental system and different exposures (2). Exposures to DMSO and/or 1NA-PP1. Each flow corresponds to a time-course as indicated in square boxes. Gravid adult animals were sacrificed to obtain synchronized populations. Synchronized animals were exposed to DMSO/1NA-PP1 during different larval stages and observed immediately or at an adult stage. Click here for file
