# Supplementary figures and images for: A chemical-genetic strategy reveals distinct temporal requirements for SAD-1 kinase in neuronal polarization and synapse formation
Source: Neural Dev. 2008 Sep 22;3:23. doi: 10.1186/1749-8104-3-23 (PMC2564922; doi:10.1186/1749-8104-3-23)

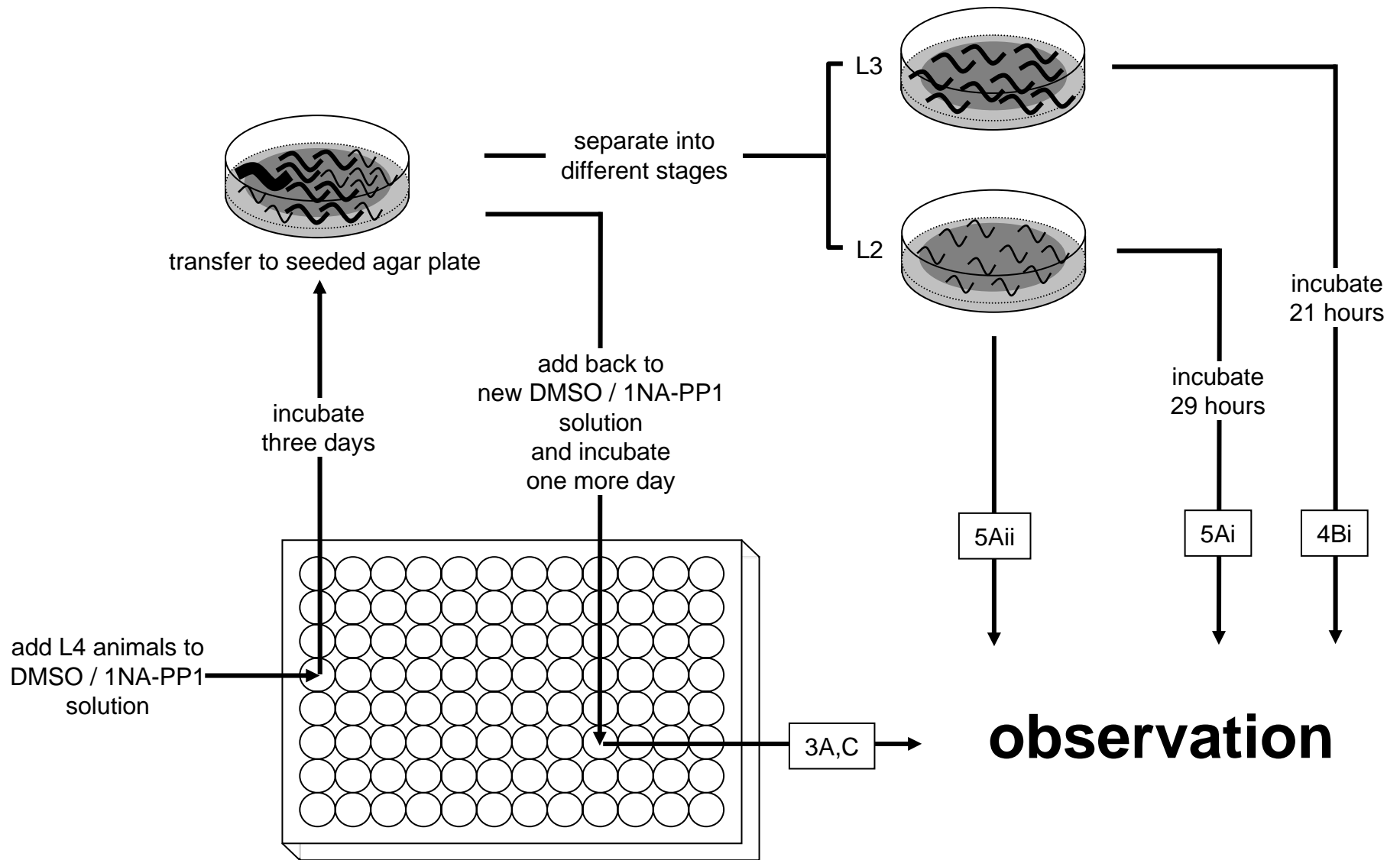

Supplement: Additional file 1 — Experimental system and different exposures (1). Exposures to DMSO and/or 1NA-PP1. Each flow corresponds to a time-course as indicated in square boxes. L4 animals were exposed to DMSO/1NA-PP1, and their progenies were transferred to agar plates at different stages to be observed immediately or at an adult stage. Full lifetime exposures required four days of incubation; solutions were renewed after three days. [file 1749-8104-3-23-S1.pdf]

transfer adult animals to hypochlorite solution  
on unseeded plate and incubate 16-18 hours

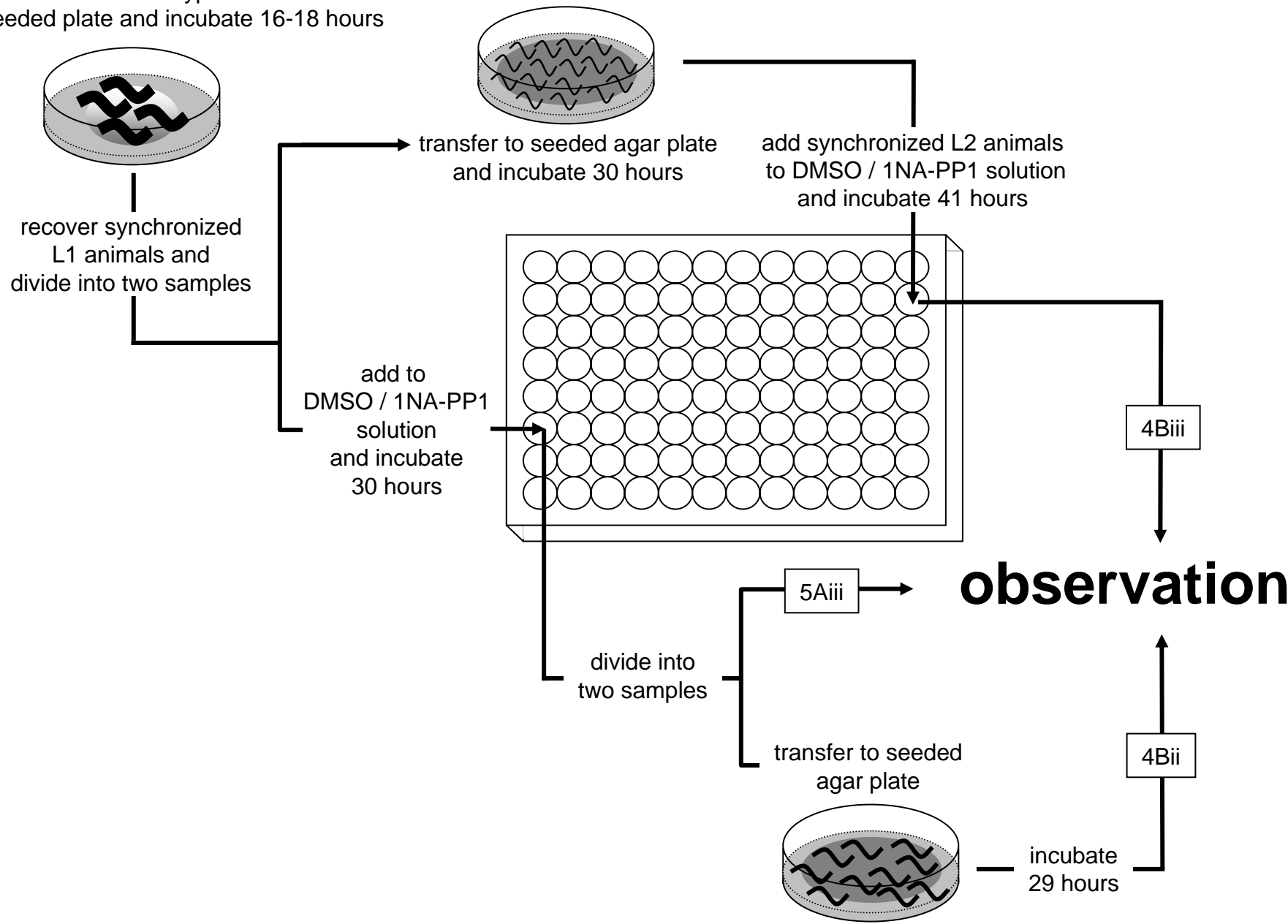

Supplement: Additional file 2 — Experimental system and different exposures (2). Exposures to DMSO and/or 1NA-PP1. Each flow corresponds to a time-course as indicated in square boxes. Gravid adult animals were sacrificed to obtain synchronized populations. Synchronized animals were exposed to DMSO/1NA-PP1 during different larval stages and observed immediately or at an adult stage. [file 1749-8104-3-23-S2.pdf]
